# Single-cell transcriptome analysis of CD8 ^+^ T-cell memory inflation

**DOI:** 10.12688/wellcomeopenres.15115.1

**Published:** 2019-05-09

**Authors:** Andrew J. Highton, Madeleine E. Zinser, Lian Ni Lee, Claire L. Hutchings, Catherine De Lara, Chansavath Phetsouphanh, Chris B. Willberg, Claire L. Gordon, Paul Klenerman, Emanuele Marchi

**Affiliations:** 1 Peter Medawar Building for Pathogen Research, Nuffield Department of Medicine, University of Oxford, Oxford, Oxfordshire, OX13SY, UK; 2 National Institute for Health Research Oxford Biomedical Research Centre, Oxford, OX42PG, UK

**Keywords:** cytomegalovirus, adenovirus vector, memory T cells, resident-memory T cells, memory inflation

## Abstract

**Background**: Persistent viruses such as murine cytomegalovirus (MCMV) and adenovirus-based vaccines induce strong, sustained CD8 ^+^ T-cell responses, described as memory “inflation”. These retain functionality, home to peripheral organs and are associated with a distinct transcriptional program.

**Methods**: To further define the nature of the transcriptional mechanisms underpinning memory inflation at different sites we used single-cell RNA sequencing of tetramer-sorted cells from MCMV-infected mice, analyzing transcriptional networks in virus-specific populations in the spleen and gut intra-epithelial lymphocytes (IEL).

**Results**: We provide a transcriptional map of T-cell memory and define a module of gene expression, which distinguishes memory inflation in spleen from resident memory T-cells (T _RM_) in the gut.

**Conclusions**: These data indicate that CD8 ^+^ T-cell memory in the gut epithelium induced by persistent viruses and vaccines has a distinct quality from both conventional memory and “inflationary” memory which may be relevant to protection against mucosal infections.

## Introduction

Persistent viruses such as murine cytomegalovirus (MCMV) and adenovirus-based vaccines induce strong, sustained CD8 ^+^ T-cell responses, described as memory “inflation” ^1^. These retain functionality and home to peripheral organs, leading to interest in these cells in vaccination ^1^.

T-cell immunity in tissues is an area of active enquiry. Murine CMV (MCMV)-specific T resident memory (T _RM_) (defined by CD69 and CD103 co-expression) has been described in the female reproductive tract, mammary gland and intestine ^2^, and examined in detail in the salivary gland (SG) where T _RM_ populations protect against local re-infection, suggesting a role against viral reactivation ^3^. Peripheral memory CD8 ^+^ T-cells (T _PM_), defined by intermediate chemokine receptor CX3CR1 expression, survey peripheral tissues, self-renew and contribute to other memory subsets ^4^. MCMV- and adenovirus vaccine-induced inflationary T-cells sustain high levels of circulating T _PM_ cells, which contribute to expanding CX3CR1 ^hi^ effector-memory populations in tissues with high levels of antigen exposure (e.g. liver, lung) ^5^. In addition, a subset of cytotoxic KLRG1 ^+^ effector memory CD8 ^+^ T-cells downregulate KLRG1 (“exKLRG1”) and differentiate into all memory T-cell lineages including CX3CR1 ^int^ T _PM_ and CX3CR1 ^neg^ T _RM_ cells ^6^.

To further define the nature of the transcriptional mechanisms underpinning memory inflation at different sites we used single-cell RNA sequencing of tetramer-sorted cells from MCMV-infected mice, analyzing transcriptional networks in virus-specific populations in the spleen and gut intra-epithelial lymphocytes (IEL). These data indicate that CD8 ^+^ T-cell memory in gut epithelium induced by persistent viruses and adenovirus vaccines has a distinct quality from both conventional and “inflationary” memory.

## Methods

### Ethics statement

All work was carried out in accordance with Animal [Scientific Procedures] Act 1986, with procedures reviewed by the clinical medicine animal care and ethical review body (AWERB), and conducted under project licence PPL 30/3293 at the University of Oxford.

### Mice

C57BL/6 mice were obtained from Harlan, UK and kept under conventional specific pathogen free (SPF) conditions in individually ventilated cages (GM500 Mouse IVC Green Line, Techniplast), fed with normal chow diet and had a standard light/dark cycle. Three to five mice were used for each experimental group, as this is the smallest number of mice that can be used to show statistically significant differences between conventional and memory populations. A single mouse was used for the single-cell RNA sequencing experiment. Conventional memory responses in the MCMV model provide an internal control for the analysis of the inflating memory response because variables such as viral replication and antigen persistence are identical. As such, an uninfected control group was not needed. One mouse was considered one experimental unit. Female mice (approx. weight 20g) were infected or immunized intravenously via tail vein injection in a biosafety cabinet class II when aged 6–8 weeks. Intravenous administration is required to develop inflationary memory T cell responses. Welfare assessments were performed daily prior to, during and after the experiment. Only health animals were used in experiments. No adverse events occurred in the experiments. All efforts were made to ameliorate any suffering of animals. MCMV infection of mice does not cause any outward signs of illness and mice continue to behave and gain weight as normal. When performing injections, time spent in the warming chamber was minimized and a new needle was used for each injection. Cages always contained environment enrichment such as sizzle and cardboard tunnels allowing mice to perform characteristic behaviors. Mice were humanely euthanized by cervical dislocation so that tissues could be immediately retrieved for processing. All efforts were made to ameliorate harm. Our experiments did not have any implications for the replacement, refinement or reduction (the 3Rs) of the use of animals in research. We used the ARRIVE checklist when writing our report ^7^

### Viruses

MCMV (Strain Smith, ATCC: VR194) was kindly provided by Professor U.H. Koszinowski, Department of Virology, Max von Pettenkofer Institute, Germany. MCMV was propagated and titrated on NIH 3T3 cells (ECACC), stored at -80°C and injected i.v. at a dose of 1 x 10 ^6^ pfus. Recombinant adenovirus expressed the βgal protein under the control of the human CMV promoter (AdLacZ ^8^; kindly provided by Dr. S. Rusconi, University of Fribourg, Fribourg, Switzerland) was propagated on HER-911 cells and purified with the Vivapure AdenoPack 20 (Sartorius, Goettingen, Germany, catalog no 14-558-548). Virus titer was determined in a cytopathic effect assay. In brief, serial dilutions of the adenovirus were used to infect HER-911 cells on a 96-well microtiter plate, and cytopathic effect was determined after 5 d by microscopy. Tissue culture infectious dose of 50% was calculated by the Reed–Muench method. AdLacZ was stored at -80°C and injected i.v. at a dose of 2x10 ^9^ pfus.

### Antibodies

We obtained the following antibodies from Biolegend (San Diego, California, USA): CD8 (clone 53-6.7) PerCpCy5.5 (catalog no 100733, rat, 1:400), CD62L (MEL-14) AF700 (catalog no 104426, rat, 1:800), CD69 (H1.2F3) BV605 (catalog no 104529, Armenian hamster, 1:200), CD103 (2E7) BV421 (catalog no 121421, Armenian hamster, 1:200) and BV510 (catalog no 121423, Armenian hamster, 1:200), CX3CR1 (SA011F11) BV421 (catalog no 149023, mouse, 1:100) and CCR9 (9B1) FITC (catalog no 129705, rat, 1:100). γδ-TCR (GL3) FITC (catalog no 561996, Armenian hamster, 1:50) was obtained from BD Biosciences (Franklin Lakes, New Jersey, USA). Granzyme B (NGZB) eF450 (catalog no 48-8898-80, rat, 1:50), and KLRG1 (2F1) eF450 (catalog no 48-5893-82, Syrian hamster, 1:100) were obtained from Life Technologies Ltd (Carslbad, California, USA).

### Lymphocyte isolation

Lymphocytes were extracted from the spleen as described previously ^9^. To obtain IELs, Peyer’s patches and fecal content were first removed from intestines. The intestines were then flushed with PBS and cut open longitudinally. To separate IELs from intestinal tissue, intestinal tissue was incubated in Hank's Balanced Salt Solution (HBSS) supplemented with 5mM EDTA, 10% FCS, 25 mM HEPES for 20 min at 200 rpm at 37°C, passed through a 40 µm nylon filter and purified by Percoll (GE healthcare, Little Chalfont, UK, catalog no 17089101) gradient centrifugation.

### Tetramer staining, intracellular staining and flow cytometric analysis

Tetramers were provided by the NIH tetramer core facility, USA, and tetramerized by addition of streptavidin-PE (BD Biosciences, catalog no 349023) or streptavidin-APC (Life Technologies Ltd, catalog no. SA1005). 1x10 ^6^ cells were stained using 50 µl PBS containing the tetramers for 20 min at 37°C. Cells were subsequently stained with mAb and live/dead fixable near-IR (Life Technologies Ltd, catalog no L34975) at 4°C for 20 min. For intracellular staining, cells were then fixed and permeabilized using the Foxp3/Transcription Factor Staining Buffer Set (Life Technologies Ltd, catalog no 00-5523-00), according to the manufacturer’s instructions. Flow cytometry was performed on a BD LSR II flow cytometer and analyzed using FlowJo (version 10.0.8r1, BD Biosciences).

### Isolation of single tetramer ^+^ CD8 ^+^ T-cells

CD8 ^+^ T-cells were enriched by negative selection using the Miltenyi Biotec GmbH CD8a ^+^ T-cell isolation kit (catalog no 130-104-075, Bergisch Gladbach, Germany) as per manufacturer’s instructions. After tetramer and surface staining, tetramer ^+^ CD3 ^+^ CD8 ^+^ single-cells were sorted in two 96-well plates using a SH800S cell sorter (Sony, Tokyo, Japan).

### Single-cell RNA sequencing

Single-cell RNA sequencing was performed as described by Picelli *et al.* ^10^. Sequencing libraries were prepared using the TruSeq dual-index sequencing primers (catalog no PE-121-1003, Illumina, San Diego, California) and paired-end sequencing was performed on the Illumina HiSeq4000 platform. All samples were spiked with ERCC RNA Spike-In Mix (catalog no 4456740, Thermo Fisher) as an internal control for sequencing (Extended data ^11^, Figure S1D).

### RNA-Seq bioinformatics and computational analyses

For standard RNA-seq analysis, the quality of Illumina reads was assessed with FastQC (Version 0.11.5) ^12^, next reads were trimmed of adapter contamination (100 nt) using Trimmomatic (Version 0.36) ^12^. Library sizes ranged from 0 to 1.2 million reads (Extended data ^11^, Figure S1C). Reads were then aligned to the mm10 reference genome using STAR (Version 2.4.1c) ^14^ as RNA-seq aligner. The reference genome was augmented with ERCC control sequences for mapping spike-in controls reads (Extended data ^11^, Figure S1D). Counting reads to genomic features (i.e. genes or exons) was performed with featureCounts (Subread, Version 1.6.1 ^15^). Cell QC filtering and pre-processing was performed using Scater (Version 1.4.0) ^16^. Principal component analysis was performed using R language (Scater package, Version 1.4.0 ^16^) on 114 cells that had >500 expressed features. After normalization of raw counts, further data analysis on transcriptomics was performed with in-house R scripts (See Extended data ^11^, Methods and Underlying Data ^17^). No batch effect between the two separate plates analyzed was detected. To minimize effects of differential gene expression due to cell-cycle stage, 16 cells were excluded as they were not in the G1 phase (Extended data ^11^, Figure S1B) using cyclone R Bioconductor package (Version 1.4.5) ^18^. In addition, cells expressing less than 1500 reads mapping to the murine genome (mm10) were excluded (9 cells excluded) leaving 89 cells for subsequent in-depth analysis. To account for differences in library size, we performed TMM normalization of gene expression using Scater ^16^. To produce a heatmap based on variance of the normalized expression values, heatmap values have been centralized (z-score). We assessed differential expression using edgeR (version 3.18.1) ^21^ and limma (Version 3.32.10) ^20^. For using limma, we transformed log(cpm) values using voom (Version 3.32.10) ^21^ to obtain gamma-distributed data. For creation of the volcano plot showing differential expression between the compartments we used a t-test with an adjusted p-value of p<0.00001. To find weighted correlation network analysis (WGCNA) modules, we picked an appropriate soft-thresholding power (β=2) for a network construction that follow a scale-free topology. By raising to a power β ≥ 1 (soft thresholding, in our case β=2) the absolute value of the Pearson correlations, or like in this case the biweight midcorrelations ^22^, to define a co-expression similarity, the weighted gene co-expression network construction emphasizes large correlations at the expense of low correlations ^23^.

### Statistical analysis of flow cytometry data

Statistical analysis was performed using GraphPad PRISM (version 6.0f, Graphpad software, Inc., La Jolla, CA). P-values for comparison of means was determined by two-tailed T test and corrected using Holm-Sidak for multiple comparisons.

## Results and discussion

**Figure 1.**
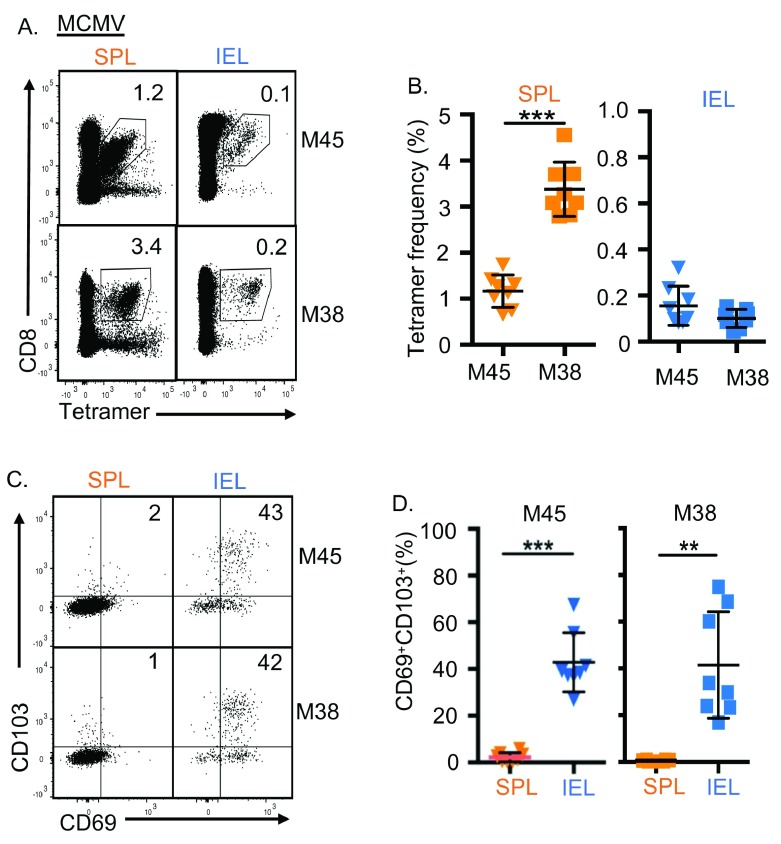
MCMV-specific CD8 ^+^ T-cells in gut intra-epithelium display features of tissue-residency. ( **A–D**) C57BL/6 mice were infected i.v. with 10 ^6^pfu MCMV, and spleen (SPL, orange), and gut intraepithelial lymphocytes (IEL, blue) sampled 28 days post infection (dpi). Data from two independent experiments is shown. ( **A**) Composite FACS plots (n=5) of M45- and M38-tetramer staining of live CD4 ^-^ lymphocytes showing mean tetramer ^+^ CD8 ^+^ T-cells (n=8). ( **B**) Mean (±SD) tetramer ^+^ CD8 ^+^ T-cells (n=8). ( **C**) Composite FACS plots (n=5) of expression of CD69 and CD103 on tetramer ^+^ CD8 ^+^ T-cells showing mean CD69 ^+^CD103 ^+^ tetramer ^+^ CD8 ^+^ T-cells (n=8). ( **D**) Mean (±SD) CD69 ^+^CD103 ^+ ^tetramer ^+^ CD8 ^+^ T-cells (n=8). p=0.05-0.011 (*), p=0.01-0.001 (**), p<0.001 (***) by t-test.

Following intravenous (i.v.) infection of C57BL/6 mice with 10 ^6^ plaque-forming units (pfu) MCMV, two distinct memory responses – a conventional contracting response (M45-tetramer ^+^) and an inflating response (M38-tetramer ^+^) - were detected in spleen and IEL ( Figure 1A-B) [2,3]. A tissue-residency phenotype (CD69 ^+^CD103 ^+^) was present in conventional and inflating CD8 ^+^ T-cell populations in IELs but not in spleen, as reported previously [2,3] ( Figure 1C-D). Residency marker expression in IELs was consistent between inflating and conventional memory T-cells ( Figure 1D), in contrast to most phenotypic markers [7].

**Figure 2.**
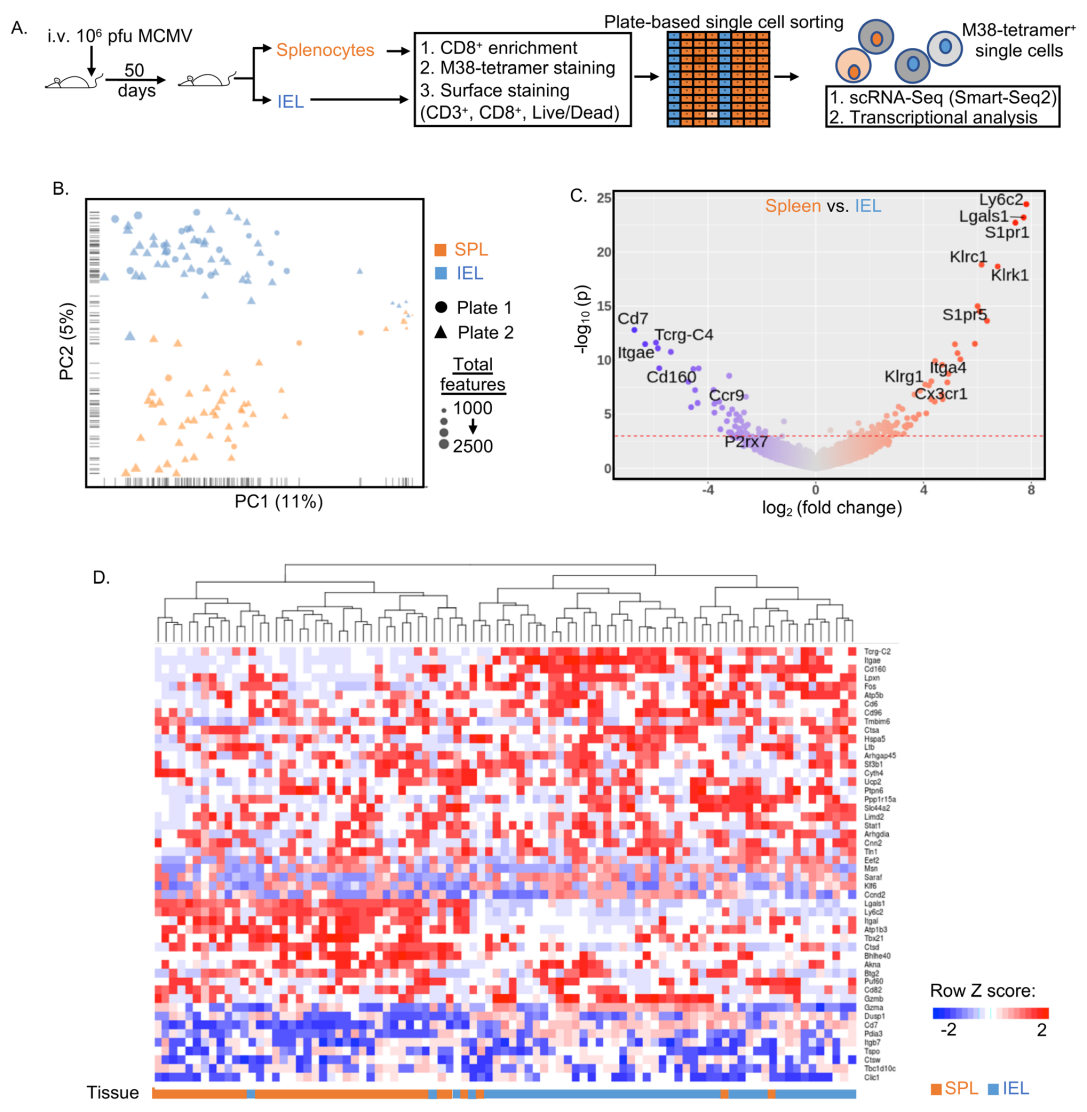
Single cell RNA sequencing reveals differences between splenic and gut intra-epithelial inflating memory cells. ( **A**) Experimental plan of single-cell RNAseq. ( **B**) 114 single M38-tetramer ^+^ CD8 ^+^ T-cells isolated from spleen or gut intra-epithelium with >500 expressed features were analyzed by PCA using the 500 most variably expressed features. ( **C**) Volcano plot of statistical significance against log2 fold-change between SPL and IEL (p<0.001; red dashed line). ( **D**) Unsupervised clustering of 50 most variably expressed genes.

**Figure 3.**
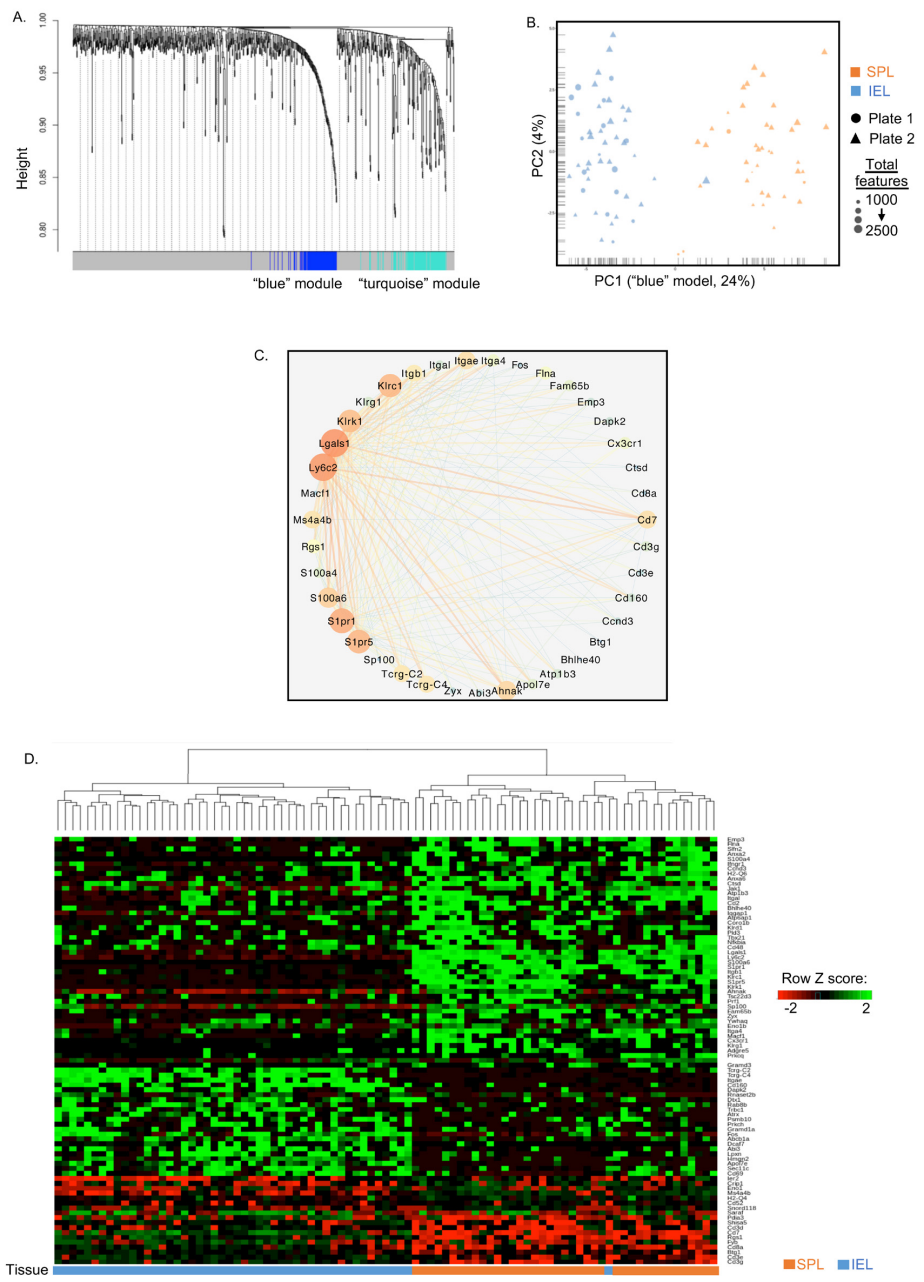
Gut intra-epithelial inflating memory cells are transcriptionally distinct from splenic inflating memory cells. Gene transcripts from 114 single M38-tetramer ^+^ CD8 ^+^ T cells isolated from spleen or gut intra-epithelium were analyzed (as in Figure 2). ( **A**) Genes were clustered into distinct modules (“turquoise” and “blue” modules) using Weighted Gene Correlation Network Analysis ^23^. The distance for the hierarchical clustering is based on a Topological Overlap Measure ^30^. ( **B**) PCA of the weighted correlation network analysis. ( **C**) Correlation network analysis using Cytoscape ^31^. Each node depicts a gene whereby the edges between the nodes show a correlative regulation of the respective genes. The thickness of the edges indicates stronger correlations between genes. The size of the node indicates the number of edges connected to the node with bigger node size results from more edges. For simplification, only nodes with an edge weight of 0.1-0.182 were used (39 genes). ( **D**) Clustering of “blue” gene module. Dendrogram shows hierarchical clustering of the dataset using Euclidian distance (right) based on genes identified in the blue gene module

To explore the transcriptional mechanisms governing memory inflation in different tissues, we performed single-cell RNA sequencing of M38-tetramer ^+^ CD8 ^+^ T-cells from spleen and gut IEL ( Figure 2A, GEO accession GSE128147). In total, 114 cells had >500 mapping features and principal component analysis (PCA) revealed compartment-dependent T-cell clustering ( Figure 2D). After excluding cells that were not in G1 cell-cycle (n=16) or had <1500 mapping reads (n=9), 89 cells were available for in-depth analysis (Extended data ^11^, Figure S1B). Normalized data was ordered by gene variance and unsupervised clustering of the 50 most variable genes revealed that the two compartments formed distinct clusters ( Figure 2D). Differential gene expression analysis ( Figure 2C; Extended data ^11^, Table S1) showed that M38-tetramer ^+^ IELs had lower expression of KLRG1 and CX3CR1 transcripts, and higher expression of integrin alpha E ( *ITGAE*, CD103) and gut-homing marker CCR9, consistent with T _RM_ cells ^24^. WGCNA ^23^ identified two gene modules (“blue” and “turquoise”) that contained highly correlated genes ( Figure 3A; Extended data ^11^, Table S2). PCA and hierarchical clustering using blue module genes clearly divided the two compartments ( Figure 3B and Figure 3D). Blue module genes are interconnected ( Figure 3C), and include genes relevant to both memory inflation (i.e. *Klrc1*, *Klrk1*(NKG2D), *Klrg1, Cx3cr1*) ^9^ and T _RM_ ( *S1pr1*, *S1pr5*, *ITGAE* (CD103) *, Klrg1*) ^24^ (Extended data ^11^, Table S2).

**Figure 4.**
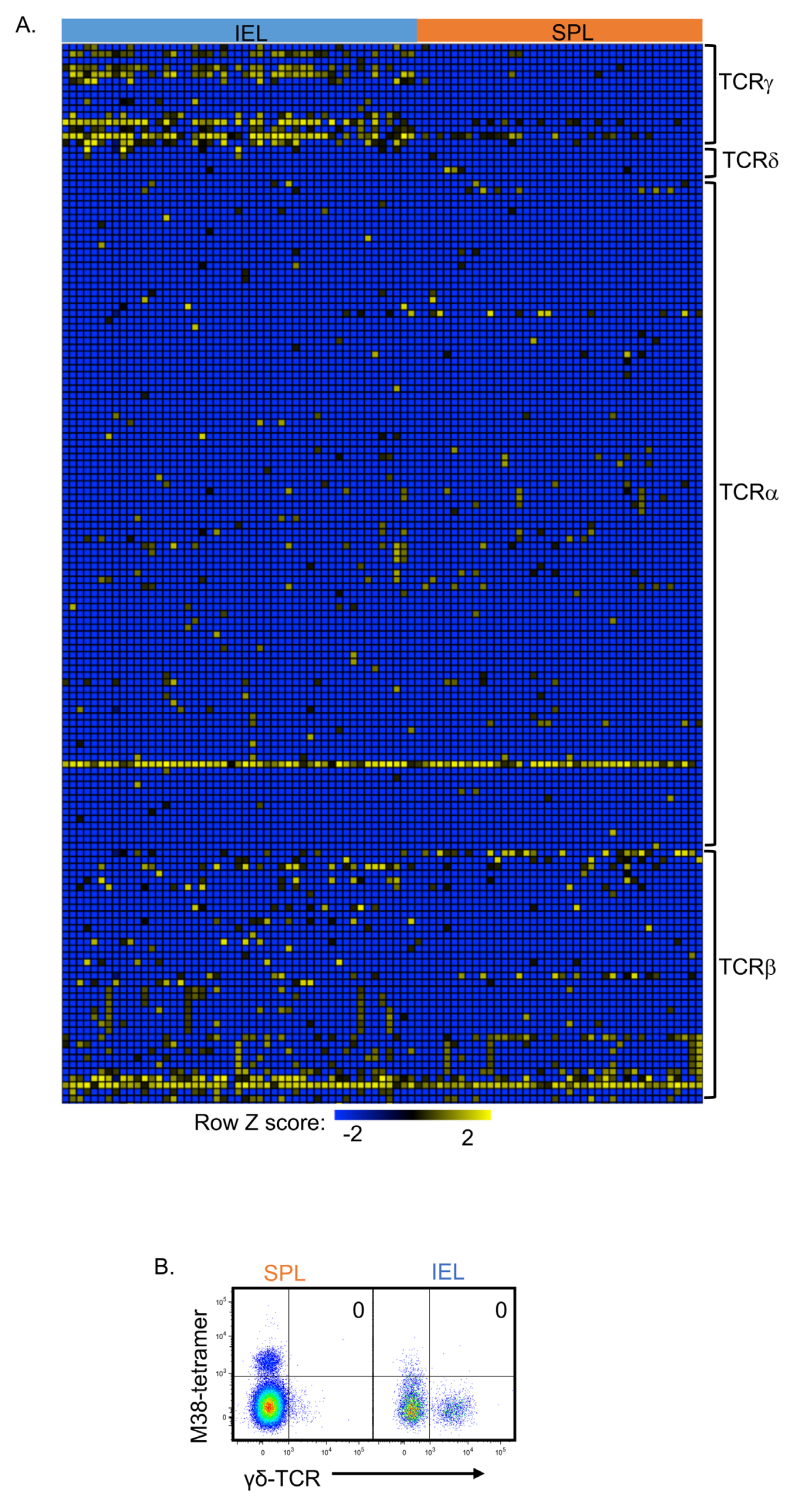
TCRγ transcripts in intestinal M38-tetramer ^+^ TCRαβ ^+^ T cells. Gene transcripts from 114 single M38-tetramer ^+^ CD8 ^+^ T cells isolated from spleen or gut intra-epithelium were analyzed (as in Figure 2 and Figure 3). ( **A**) Heatmap showing normalized expression values of TCR chain transcripts in single cells from indicated tissue. ( **B**) Representative FACS showing expression of TCRγδ and M38-tetramer staining on splenic and IEL live CD8 ^+^ T cells.

We observed differences in transcripts aligning to the constant part of the T-cell receptor (TCR) gamma chain (Tcrg-C4) ( Figure 4A), as noted before ^25^. We detected TCRγ (but not δ) transcripts in intestinal M38-tetramer ^+^ TCRαβ ^+^ T-cells but not in spleen ( Figure 4A). TCRγδ was not detected by flow cytometry on M38-tetramer ^+^ TCRαβ ^+^ T-cells from either site ( Figure 4B). Cytokine signaling via STAT5 can induce γ-chain germline transcription ^26^ – this is of interest given the role of IL-15 in tissue maintenance of memory inflation ^27^, and T _RM_ survival ^28^. 

We verified our transcriptional results and showed enhanced CCR9 and reduced KLRG1 and CX3CR1 expression on inflating IELs compared to spleen (Extended Data ^11^ Figure S3C-D). This is in contrast to circulating and lung inflationary cells that express high levels of CX3CR1 and KLRG1 ^5, 9^, likely in response to high-level antigen exposure at these sites. This discrepancy led us to hypothesize that gut-localized inflating memory cells are ex-KLRG1 ^6^. In keeping with this, gut inflating memory cells were more cytotoxic (as measured by granzyme B) than spleen (Extended data ^11^, Figure S3C-D). Tissue-specific differences in marker expression were not observed in the conventional (M45-tetramer ^+^) memory population (Extended data ^11^, Figure S3A-B).

Intravenous vaccination with a replication-deficient recombinant HuAd5 vector expressing lacZ (Ad-lacZ) induces phenotypically, functionally and transcriptionally similar inflationary responses to MCMV infection ^9^. Immunization with Ad-lacZ induced inflating (D8V-tetramer ^+^) and conventional (I8V-tetramer ^+^) memory responses in spleen and gut (Extended data ^11^ Figure S4A-B) ^9^. Similar to MCMV, inflating and conventional vaccine-derived T-cells in gut epithelium co-expressed CD103 and CD69, had low KLRG1 and CX3CR1 expression, and high granzyme B expression (Extended data ^11^, Figure S4). These data were obtained from a single experiment and needs to be validated further. These data suggest that adenoviral vectors may prime similarly regulated T _RM_ populations in the gut. Whether these results apply to CMV infection and adenovirus vector vaccination in humans remains to be determined.

Our data support a model of intraepithelial cells being “activated yet resting” ^29^: antigen-specific cells are primed during active infection and seed tissues at an early stage before the resolution of the active infection acquiring tissue-residency marker expression. Divergence at this stage is consistent with a lack of further differentiation down the inflationary pathway which is typically driven by repetitive antigen encounter. These data are consistent with mechanistic experiments performed in SG, where development of a resident memory phenotype amongst CD8 ^+^ T-cells occurs in an antigen-independent manner ^3^. Two relevant markers are CX3CR1 and KLRG1, highly expressed on inflationary CD8 ^+^ T-cells ^5, 9^ but not on T _RM_ cells ^4, 6^. The inflationary pool gives rise to CX3CR1 ^int^ T _PM_ CD8 ^+^ T-cells in early memory, which are partially differentiated but retain self-renewal potential ^4, 5^. The CX3CR1 ^int^ T _PM_ population is enriched with exKLRG1 cells ^6^. Putting it all together, we propose a model where CX3CR1 ^int^ T _PM_ seed tissues early in infection before converting to CX3CR1 ^hi ^effector-memory cells in tissues with high antigen exposure or to CX3CR1 ^neg^ ex-KLRG1 T _RM_ cells in tissues with no/low antigen exposure.

## Conclusions

We addressed the transcriptional underpinning of inflationary memory in two sites and found that IEL-associated responses showed a marked diversion away from the standard pathway of “inflation” *in vivo*. These hard-to-access antigen-specific cells were analyzed transcriptionally on a single-cell level, which revealed a distinct module closely associated with development of a gut IEL-type phenotype. In conclusion, these data indicate that CD8 ^+^ T-cell memory in the gut epithelium induced by persistent viruses and vaccines has a distinct quality from both conventional memory and “inflationary” memory which may be relevant to protection against mucosal infections.

## Data availability

### Underlying data

Single-Cell Transcriptome Analysis Of CD8+ T-cell Memory Inflation, Accession number GSE128147: https://identifiers.org/geo/GSE128147

Figshare: Flow Cytometry Data for “Single-Cell Transcriptome Analysis Of CD8+ T-cell Memory Inflation”. https://doi.org/10.6084/m9.figshare.7834880.v1 ^32^

This project contains the following underlying data:

- FACS data single cell paper.xlsx (Flow cytometry data)

Figshare: scRNASeq R pipeline for "Single-cell transcriptome analysis of CD8+ T-cell memory inflation". https://doi.org/10.6084/m9.figshare.8044736 ^17^

This project contains the following underlying data:

- scRNASeq_pipeline.R (scRNASeq R pipeline)
- Andy_Mad_tcr_profile.R (R script for T cell receptor analysis)

### Extended data

Figshare: Extended data is deposited in Figshare: Extended Data for “Single-Cell Transcriptome Analysis Of CD8+ T-cell Memory Inflation”. https://doi.org/10.6084/m9.figshare.7666418 ^11^

This project contains the following extended data:

- Extended data WOR.pdf (Additional figures and tables)

Data are available under the terms of the Creative Commons Attribution 4.0 International license (CC-BY 4.0).
