# Supplementary material for: Single-cell transcriptome analysis of CD8 + T-cell memory inflation
Source: Wellcome Open Res. 2019 May 9;4:78. [Version 1] doi: 10.12688/wellcomeopenres.15115.1 (PMC6688724; doi:10.12688/wellcomeopenres.15115.1)
Supplement: Peer review history

# Peer review: Reviewer response for version 1

**DOI:** 10.21956/wellcomeopenres.16491.r35918

**Published:** 2019-08-08

**Authors:** Birgit Sawitzki

**Affiliations:** 1 Institute of Medical Immunology, Charité – Universitätsmedizin Berlin, Berlin Institute of Health, Free University of Berlin, Humboldt University of Berlin, Berlin, Germany

Within this manuscript Highton and colleagues provide a very interesting first report on transcriptional differences of inflationary MCMV-specific CD8+ memory accumulating in spleen or in the gut epithelium.

 Interestingly, gut intra-epithelial cells (IEL) inflational memory T cells were characterised by dramatically reduced KLRG1 and CX3CR1 expression but showed higher CCR9 expression compared to their splenic counterparts. This is in striking contrast to previous findings on lung resident inflationary memory T cells, which show very high CX3CR1 and KLRG1 expression. This might point to tissue-specific differences in transcriptional profiles and not related to different infectious agents as also IEL inflational memory induced upon adenoviral vaccination showed reduced CX3CR1 and KLRG1 expression. With this phenotype IEL-resident inflational memory T cells resemble previously described exKLRG1 cells, which can convert into CX3CRhighKLRG1+ effector T cells in case of high antigen encounter.

 Especially the overlap between MCMV- and vaccination-induced memory T cell transcriptional profiles are of major interest as they highlight the potential of adenovirus-based vaccines to induce long-term functional tissue resident memory.

 Clearly, the study leaves several open questions, but it is very important starting point for future investigations.

 While reading the manuscript a few specific questions arose:

1. Vaccination-induced gut IEL inflational memory T cells do not show CCR9 and Granzyme B expression, which might influence protective capacity and functionality as previously described. Is that related to the infectious agent or timing (kinetics) as priming upon adenoviral vaccination takes longer?
2. Can gut IEL memory T cells be further subdivided according to e.g. CCR9, Granzyme B and CD160 co-expression defining different functional states? This would be really interesting as conventional IEL memory T cells seem to be nearly negative for CCR9 and Granzyme B and thus potentially less protective.

I confirm that I have read this submission and believe that I have an appropriate level of expertise to confirm that it is of an acceptable scientific standard.

# Peer review: Reviewer response for version 1

**DOI:** 10.21956/wellcomeopenres.16491.r35919

**Published:** 2019-07-30

**Authors:** Charles H. Cook

**Affiliations:** 1 Beth Israel Deaconess Medical Center, Harvard Medical School, Boston, USA

This work adds another interesting piece to our understanding of virus specific T-cells after CMV infection. While a biostatistician might laugh a little at my interpretation, I am impressed that unsupervised clustering allowed almost complete resolution between the splenocytes and IEL. With further subset analysis (blue/turquoise, and forgive my characterization if incorrect here) it seems possible to easily distinguish between the two populations. Thus I am persuaded that the virus specific T-cells from these two different sites seem clearly different in numerous ways. I have only a few concerns that if addressed I think will enrich the manuscript.

 Concerns:

1. I am a little confused by the development of gut IELs in this system. None of the infections were enteral, and current data for CMV support a oropharyngeal/pulmonary model as the more likely “natural” route of infection anyway. So why do these virus specific cells migrate to the gut and hang out? Is there precedent for this in other infection models – i.e. is this just something that the body naturally does in response to most/all infections? The adenovirus experiments shown actually support that this is the case. There is an opportunity missed here to discuss this possibility particularly vis a vis other pathogens.
2. I think that the authors are probably correct in their assumption that virus specific IEL are not encountering antigens given the phenotypic differences shown. This is also consistent with the significant differences in TCR demonstrated. Do the current observations align with recent data from Nikolich Zugich on TCR receptor evolution over time?
3. There is no mention of CD8 T-cells isolated from other sites, such as the lung. One might expect that given their association with the circulation and not tissue, lung T-cells might show a similar phenotype to splenocytes. If this has been looked at and there is no difference that would warrant mention here I think. If there are differences then I suppose I will be waiting with bated breath to read about those differences later.

Minor concerns

1. Figure s2 actually illustrates the differences in T-cell receptor usage better than Figure 4A - I think you should consider swapping these.
2. Table s1 is sorted by p-value… I would be more interested in seeing this sorted differently. By marker to facilitate locating a specific marker would be best I think, or by fold change to give a better impression of what was more highly up or down regulated.

I confirm that I have read this submission and believe that I have an appropriate level of expertise to confirm that it is of an acceptable scientific standard.
